# P26 A case of B-cell lymphoma affecting the eye in a patient with rheumatoid arthritis following a reduction in the frequency of rituximab therapy

**DOI:** 10.1093/rap/rkab068.025

**Published:** 2021-10-19

**Authors:** Yen Zi Lau, Ryan Malcolm Hum, Pauline Ho

**Affiliations:** 1 School of Medicine, University of Nottingham Medical School, Nottingham, United Kingdom; 2 The Kellgren Centre for Rheumatology, Manchester University NHS Foundation Trust, Manchester, United Kingdom; 3 Centre for Musculoskeletal Research, Faculty of Biology, Medicine and Health, The University of Manchester, Manchester, United Kingdom

## Abstract

**Case report - Introduction:**

Rheumatoid arthritis (RA) is a life-long systemic autoimmune inflammatory disease associated with numerous co-morbidities, one of which includes the increased risk of developing lymphoproliferative disorders. RA patients have been found to be at increased risk of developing lymphoma, with non-Hodgkin’s lymphomas (NHL), especially B-cell lymphoma, being the most common. Rituximab is a biologic disease modifying anti-rheumatic drug (bDMARD) that inhibits b cells used in oncology and rheumatology. In this case report, we present a middle-aged female with RA who developed B-cell lymphoma affecting her right eye after a reduction in the frequency of her rituximab therapy.

**Case report - Case description:**

A 43-year-old female was diagnosed with RA at age 27 and experienced dry eyes and corneal ulcers during her disease. Her RA was well controlled on adalimumab.

She presented to clinic with right-sided eyelid and lacrimal gland swelling which had progressed over 4 months, with reduced eye motility and visual acuity (6/24). A CT scan revealed a mass lesion in the superolateral quadrant of the orbit involving the lacrimal gland. Histology from an incisional biopsy concluded that the diagnosis was “chronic idiopathic orbital inflammation”. The immunohistochemical report revealed a mixture of B- and T-cell populations, with no definite light chain restriction. The test for serum IgG4 was negative, ruling-out IgG4 disease. She was subsequently admitted to the hospital and received three 250mg infusions of methylprednisolone, followed by 6-monthly rituximab infusions. Following methylprednisolone therapy, her vision had improved 6/6 on the right, and 6/5 on the left. She went on to respond to the rituximab treatment, and the orbital mass shrank as her vision returned to baseline.

After 7 years on 6-monthly rituximab cycles, her RA remained stable. Her immunoglobulins were normal, and she requested to be transferred to a local hospital for convenience of travel. At this centre, she received her rituximab infusions only when her disease would flare. After several years on this new regime frequency, she only received 1 treatment in 14 months when she presented with a flare in ocular symptoms. She presented with 2mm proptosis, and ptosis of the right eyelid, alongside a rapidly growing subcutaneous mass on her forehead. The mass was biopsied and determined to be a high-grade B-cell lymphoma. Following 12 months of chemotherapy, radiotherapy, and an autologous stem cell transplant, she went into remission. Her rituximab therapy was changed back to 6-monthly, and her RA remains stable with no further ocular complications.

**Case report - Discussion:**

RA is associated with an increased risk of cancer; however, the aetiopathogenesis is unclear, though the role of chronic inflammation has been reported. A study by Wolfe et al. found that in patients with chronic inflammation, the risk of developing NHL was 9-times higher. Similarly, a Swedish study following 378 RA patients and 378 control patients reported that in patients with severe longstanding RA, the risk of developing cancer was significantly greater in those with higher disease activity.

Furthermore, the role of immunomodulatory agents such as rituximab in altering the risk of malignancy is unclear. Rituximab inhibits B-cell activity, which may play a role in supressing the development of B-cell malignancies. A review of the literature found no studies about rituximab and reduced incidence of lymphoma in RA specifically; however, several studies in other diseases have reported a reduction in the incidence of lymphoma in patients taking rituximab. A study by Gérard et al. followed 113 patients with HIV-associated multicentric Castleman disease for 15 years, where 48 patients were given rituximab therapy and 65 patients were not. Only one patient in the rituximab cohort developed NHL, whereas 17 patients in the other cohort developed NHL. In another study using patient-derived xenografts, immunocompromised mice with high susceptibility of lymphoproliferative disease were implanted with gastric carcinomas. Over 30% of them developed human B-cell lymphomas; however, they found that if the mice were injected with rituximab upon implantation, lymphoma would cease to develop.

**Case report - Key learning points:**

Several studies have demonstrated that rituximab therapy plays a significant role in reducing the incidence of lymphoma. In our case report, the development of lymphoma coincided in a reduction in the frequency of rituximab therapy. Therefore, it is possible that the loss of the suppressive effect of rituximab in our patient played a role in the development of lymphoma.

Data from real-world drug registries such as the British Society of Rheumatology Biologics Registry for Rheumatoid Arthritis (BSRBR-RA) could provide insight into the incidence and age of onset of B-cell malignancies in patients taking rituximab, compared to those taking non-B-cell-modulating therapy. Similarly, these data could provide insight into whether this protective effect also applies to T-cell-modulating therapy and the risk of T-cell malignancies.

Finally, rituximab was also integral in managing the ocular manifestations of her disease. Therefore, this case reinforces that rituximab remains a good treatment option for RA patients with ocular involvement.

In conclusion, rituximab is an effective treatment option in managing the ocular manifestations of RA, and may play a role in suppressing the development of lymphoma.

